# Protons or Photons in Pituitary Neuroendocrine Tumors—That Is Not the Question

**DOI:** 10.1016/j.ijpt.2025.101194

**Published:** 2025-06-18

**Authors:** Liv Cathrine Heggebø, Frank Leonel Bello Garrote, Lars Fredrik Fjæra, Maziar Hervani, Christina Ramberg, Andreas Ottestad, Hanne Blakstad, Trine Martens, Corina S. Rueegg, Magnus Gustavsson, Thomas Henry, Malin Blomstrand, Taran Paulsen Hellebust, Petter Brandal

**Affiliations:** 1 Department of Oncology, Oslo University Hospital, Oslo, Norway; 2 Institute of Clinical Medicine, University of Oslo, Oslo, Norway; 3 Department of Medical Physics, Oslo University Hospital, Oslo, Norway; 4 Oslo Centre for Biostatistics and Epidemiology, Oslo University Hospital, Oslo, Norway; 5 Department of Medical Physics and Biomedical Engineering, Sahlgrenska University Hospital, Gothenburg, Sweden; 6 Department of Medical Radiation Science, Institute of Clinical Sciences, Sahlgrenska Academy, Gothenburg, Sweden; 7 Department of Oncology, Sahlgrenska University Hospital, Gothenburg, Sweden; 8 Department of Oncology, Institute of Clinical Sciences, Sahlgrenska Academy, University of Gothenburg, Gothenburg, Sweden; 9 Faculty of Health and Social Sciences, University of South-Eastern Norway, Oslo, Norway; 10 Institute for Cancer Genetics and Informatics, Oslo University Hospital, Oslo, Norway

**Keywords:** Pituitary neuroendocrine tumors, Proton therapy, Radiation therapy, Treatment planning, Plan comparison

## Abstract

**Purpose:**

With the long-life expectancy of patients with pituitary neuroendocrine tumors (PitNETs), reducing long-term toxicity is essential to preserve quality of life. We aimed to investigate whether 2- or 3-field proton beam therapy (PBT) plans better spare organs at risks (OARs), including healthy tissue, than volumetric modulated arc photon therapy (VMAT).

**Materials and Methods:**

Fifteen consecutive patients who had received radiation therapy for PitNETs were included. We made 4 treatment plans for each patient: one 3-field PBT plan (3F), two 2-field PBT plans (2F-L and 2F-IV), and 1 VMAT plan. Dosing was set to 1.8 Gy × 30 to allow for dosimetric comparisons.

**Results:**

All 4 treatment techniques showed excellent target volume coverage. Compared to VMAT, the 3 PBT techniques had lower doses to OARs. Median D_40%_ to the hippocampi varied from 0.4 to 1.1 Gy (relative biological effectiveness [RBE]) with PBT and was 5.2 Gy for both hippocampi for VMAT. Median mean dose to brain-CTV and head-CTV were lowest for 2F-L (3.1 and 2.3 Gy [RBE]), followed by 3F (3.9 and 2.5 Gy [RBE]), 2F-IV (4.1 and 2.6 Gy [RBE]), and highest for VMAT (4.8 and 4.7 Gy). A relative reduction in integral dose compared to VMAT was seen for all PBT techniques, with a median reduction of 38.5% to 45.9% for head-CTV.

**Conclusion:**

Proton techniques spared OARs better than VMAT; however, absolute dose sparing was relatively small, and the clinical value remains uncertain. Nonetheless, the dose reduction, especially for the hippocampi and integral dose, is probably clinically meaningful. We argue that head-CTV should be assessed, as much of the volume receiving dose is located outside the brain. The size and location of the target volume impacted which proton plan was preferable. We conclude that individualized planning is more important than a mere question on protons or photons in PitNET patients.

## Highlights

• Head-CTV should be assessed for pituitary neuroendocrine tumors (PitNETs) patients.
• Proton techniques lead to considerable integral dose sparing in Pit-NET patients.
• Individualized proton dose planning is crucial in patients with Pit-NET.

## Graphical abstract

**Figure:**
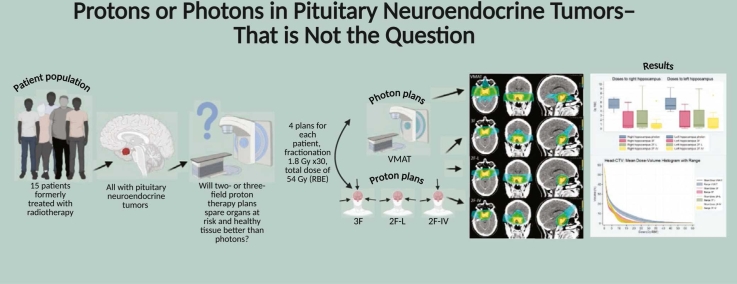

## Introduction

Pituitary neuroendocrine tumors (PitNETs) are classified according to the World Health Organization classifications of central nervous system tumors^1^ and of pituitary tumors.^2^ PitNETs account for 10% to 17% of primary brain neoplasms.^3^, ^4^ Incidence has been increasing in the last decades, supposedly mainly due to increased magnetic resonance imaging availability, and there are now approximately 5 cases diagnosed per 100 000 individuals.^4^, ^5^ Interestingly, a systematic review found an estimated prevalence of PitNETs in the general population of 16.7% based on radiographic and postmortem studies.^6^

Antineoplastic treatment aims to halt tumor growth, normalize hormone secretion, and/or prevent problems related to mass effects without harming the patient.^7^ Radiation therapy is indicated in cases of inoperable large residual or recurrent symptomatic PitNETs that do not respond to medical treatment.^3^, ^8^ Radiation therapy for PitNET patients yields excellent progression-free survival rates of 80% to 97% (10-year) and 75% to 96% (20-year), and overall survival rates of 74% to 93% at 10 and 49% to 61% at 20 years.^9^, ^10^, ^11^, ^12^ The most common late effect following radiation therapy for PitNETs is hypopituitarism,^9^, ^13^, ^14^ with increasing risk over time, from 20% 5 years post radiation to about 80% 10 to 15 years post radiation.^13^, ^14^ Other adverse effects include cognitive impairment, fatigue, radiation-induced optic neuropathy, secondary neoplasms, and cerebrovascular events.^10^, ^15^, ^16^, ^17^, ^18^, ^19^, ^20^, ^21^, ^22^, ^23^, ^24^

With the long life expectancy of PitNET patients, reducing long-term treatment-related toxicity is essential to preserve quality of life.^7^ Stereotactic radiosurgery is often preferred for PitNETs measuring <2.5 to 3.0 cm with a safe distance to the anterior optic pathways, whereas fractionated radiation therapy is favored for larger tumors and lesions kissing the anterior optic pathways (12, 13). Proton beam therapy (PBT) has an advantageous energy deposition, and for patients with brain and skull base lesions, the dose to organs at risk (OARs) will be lower compared to photon radiation therapy.^25^, ^26^, ^27^, ^28^, ^29^ Furthermore, reducing the integral dose to healthy brain tissue is one of the greatest advantages of proton therapy, likely reducing the risk of long-term complications, such as secondary neoplasms.^21^, ^30^, ^31^, ^32^, ^33^ However, dosimetric studies on PitNET patients are limited, and comparisons have evaluated older proton delivery systems (passive scatter) and not up-to-date treatment planning techniques with robust optimization.^25^, ^26^, ^34^ Moreover, the use of the passive scatter technique may explain the fact that clinical studies have failed to show the superiority of PBT for PitNET patients.^8^, ^35^, ^36^, ^37^ Therefore, dosimetric comparison using modern photon and proton treatment techniques for PitNET patients is warranted. In the present study, we aimed to investigate to what degree OARs and healthy tissue can be spared using PBT employing pencil beam scattering, robust optimization, and different field arrangements compared to photon radiation therapy.

## Materials and methods

The Radiotherapy Treatment plannING study Guidelines framework^38^ and the format for dosimetry comparison studies suggested by Amdur et al^39^ were applied when conducting this study.

### Patients

Fifteen consecutive patients treated for PitNETs between 2015 and 2017 were included. Tumor size at the time of radiation therapy ranged from 11 × 9 × 8 to 39 × 27 × 25 mm. Ten patients had nonsecreting adenomas, and all 15 had undergone surgery before radiation therapy (Supplementary Table 1). The study was approved by the institutional data protection office.

### Target volume and organ at risk delineation

All patients’ OARs were redelineated according to today’s standard practice.^40^ In addition to standard in-house OARs, the suprasellar cistern (SC) was delineated as a surrogate for the circle of Willis (CW) and the large intracranial arteries.^19^, ^20^, ^41^ Computed tomography (CT) scans with slice thickness ranging from 1 to 3 mm were used for treatment planning. The patients were fixated with a thermoplastic mask with the patient’s neck in a neutral position. CT scans were merged and matched with dose-planning magnetic resonance imaging series. The original gross tumor volume and clinical target volume (CTV) were retained, including the originally used 5 mm margin from gross tumor volume to CTV, adjusted for anatomical boundaries and air. Head-CTV was standardized for all patients as the volume defined by the external contour (fixation equipment not included), cranially from the top of the skull to caudally the dorsal, caudal barrier of the dens axis (C2), excluding CTV. Planning risk volume for photon plans was generated by adding a 2 mm margin from delineated OARs.

### Radiation therapy dose and planning techniques

Treatment plans were prepared with a total dose of 54.0 Gy in 30 fractions prescribed to the CTV median dose to allow for dosimetric comparisons. Assuming a relative biological effectiveness (RBE) of 1.1, doses from proton treatment plans are expressed in Gy (RBE). All plans were generated in RayStation 12A SP1. Coverage of target volumes, dose conformity, and OAR restrictions for brainstem, chiasm, and optic nerves were prioritized, followed by doses to other OARs. Dose constraints to OARs followed the EPTN consensus^42^ and national standard clinical practice.^43^ Doses as low as reasonably achievable to all OARs were strived for.

#### Photon planning

For photon plans, the institutionally used margin of 2 mm was added from CTV to define the planning target volume, aiming at covering ≥98% of the planning target volume with 95% of the prescribed dose. We applied volumetric modulated arc therapy (VMAT) for photon planning. In most cases, 2 full arcs with the collimator at 30°/330° were used. Photon calculations were performed with a beam quality of 6 megavoltage on a Varian TrueBeam STx equipped with a 120 multileaf collimator, and a resolution of 2.5 mm at the isocentre for the central 8.0 cm and 0.5 mm in the outer region. One experienced dosimetrist prepared all 15 VMAT plans.

#### Proton planning

**Figure 1:**
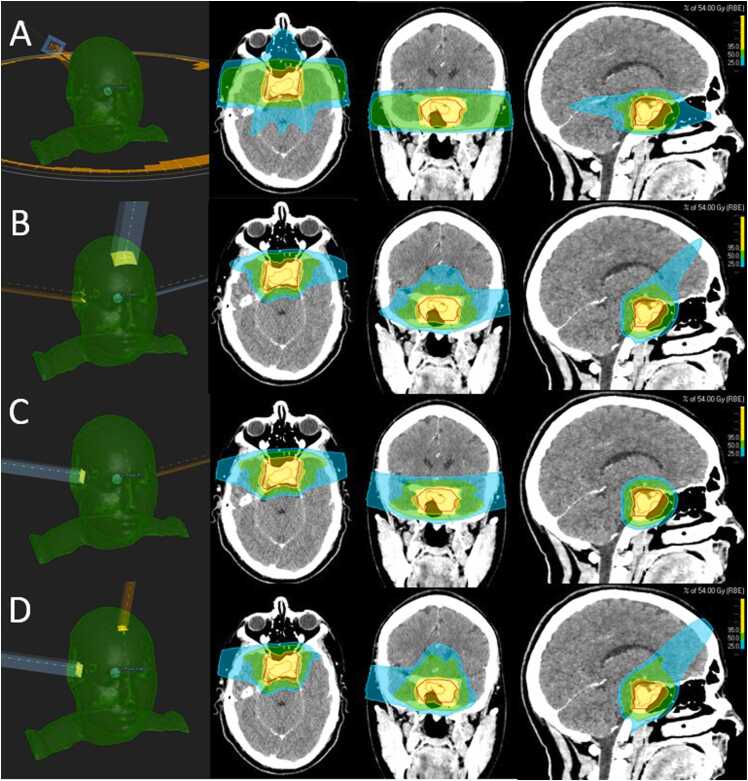
Radiation therapy plans from 1 patient comparing Volumetric Modulated Arc Therapy (VMAT) (A), 3-field proton (B), 2-field proton with 2 lateral fields (2F-L) (C), and 2-field proton with 1 ipsilateral and 1 vertex field (2F-IV) (D). The left column demonstrates field angles for the different treatment techniques (D) is an example with the lateral field coming from the patient’s right side. Gross total volume (GTV) is delineated in orange and clinical target volume (CTV) in red. The blue color represents 25%, green 50%, and yellow 95% of the prescribed dose of 54 Gy (RBE) (relative biological effectiveness).

For all 15 cases, 3 intensity-modulated proton therapy plans were made using a Varian ProBeam 360 beam model, 1 with 3-field (3 F) and 2 with different 2-field techniques (one with opposed lateral fields [2F-L] and one with 1 ipsilateral and 1 vertex field [2F-IV])—all using multifield optimization (Figure 1). For the 2F-L plans, the fields were angled anteriorly to avoid the hippocampi. Furthermore, a couch kick was utilized to prevent alignment with the skull base. For the 3F plans, a vertex field was added, and caution was exercised to ensure the dose was not sent deep into the pharynx. Similar caution was taken for the vertex field in the 2F-IV plan, and the direction of the lateral field was chosen based on the lateralization of each patient’s target volume. Among the 2F-IV plans in this cohort, 7 plans had a field from the left side and 8 from the right. These 3 PBT field set-ups were applied to investigate whether a likely clinical dose sparing of contralateral OARs was seen using the 2F-IV technique, compared to 2F-L and possibly also the 3F techniques. For all 45 plans, robust optimization and evaluation were performed using a ±2 mm patient position uncertainty and a density uncertainty of ±3.5%. Robust evaluation included diagonal shifts and no shifts, resulting in 30 scenarios. The 95% isodose should cover ≥98% of the CTV in the worst-case scenario. The RayStation Monte Carlo algorithm was used for optimization and final dose calculation. Four different planners made the 45 plans (2 dosimetrists and 2 medical physicists).

### Evaluation

All 60 plans were reviewed by members of the core study group, consisting of dosimetrists, medical physicists, and radiation oncologists. Evaluation was done qualitatively and quantitatively, reviewing plans slice-by-slice and looking at physical parameters calculated in RayStation. Target volume coverage and dose constraints to OARs were evaluated for each plan, using established international dose constraints.^42^, ^43^ Maximum dose (D_0.03cm^3^_) was defined as the minimum dose in the highest exposed 0.03 cubic centimeters (cm^3^) volume and should not exceed 107% of the prescribed dose. D_99%_ (dose to 99% of the target volume) represented minimal nominal dose coverage. In addition, we calculated the relative brain-CTV volume receiving ≥30 Gy (V_30 Gy_) and the mean dose (D_mean_) to the brain-CTV volume, as these parameters are used for patient selection to proton therapy at some centers.^44^ Furthermore, we also focused on head-CTV because much of the radiation dose spilling when treating PitNETs is to structures outside the brain. The integral dose for brain-CTV and head-CTV for all 60 plans was calculated using a script made for RayStation. For the SC, D_mean_ and D_0.03cm^3^_ were evaluated.^20^, ^41^ Lastly, we calculated the dose that was received by 40% (D_40%_) of each hippocampus.

### Statistical methods

Statistical analysis was performed using Stata version 18 (StataCorp LLC, Texas, USA). We used descriptive statistics with median, minimum, and maximum values to describe doses to the different OARs. Furthermore, a post-hoc pair-wise comparison was applied to investigate whether the 4 plans differed (VMAT chosen as reference), defined as significant if 0.05 divided by 6 comparisons was <0.008. As this is an exploratory study with a small sample, no adjustment for multiple testing was performed, and the results are considered exploratory.

## Results

All 60 treatment plans had excellent target volume coverage and dose conformity, as exemplified in Figure 1. All 45 PBT plans were evaluated as acceptable using robust evaluation based on the defined number of scenarios and high-pass criteria.

**Figure 2:**
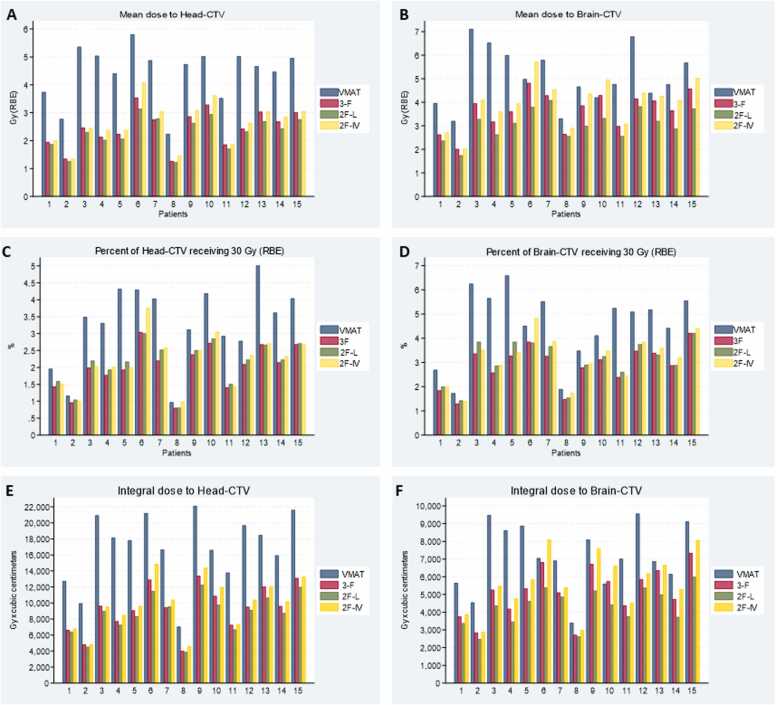
Graphs for all 15 patients comparing the 4 treatment techniques illustrating mean dose to head-CTV (A), mean dose to brain-CTV (B), the percent of head-CTV (C), and brain-CTV (D) receiving 30 Gy or more, and integral dose in Gy × cm^3^ to head-CTV (E) and brain-CTV (F). Abbreviations: 2F-IV, 2-field proton technique with 2 lateral fields; 2F-L, 2-field proton technique with 2 lateral fields; 3F, 3-field proton technique; CTV, clinical target volume; Gy, gray; RBE, relative biological effectiveness; VMAT, volumetric modulated arc therapy.

**Figure 3:**
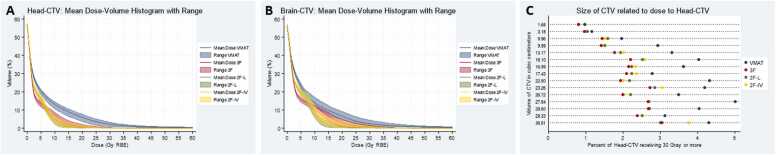
Mean dose-volume histogram curves for head-CTV (A) and brain-CTV (B) for the 4 treatment techniques. Figure (C) illustrates the relationship between CTV size and dose to head-CTV for all 15 patients. 2F-IV, 2-field proton technique with 2 lateral fields; 2F-L, 2-field proton technique with 2 lateral fields; 3F, 3-field proton technique; CTV, clinical target volume; Gy, gray; RBE, relative biological effectiveness; VMAT, volumetric modulated arc therapy.

The median V_30 Gy_ for brain-CTV and head-CTV was lowest for the 3F PBT technique, closely followed by 2F-L and 2F-IV, and highest for photons (Table 1 and Figure 2). Mean doses to brain-CTV and head-CTV were lowest for the 2F-L technique, followed by the 3F and 2F-IV techniques, and with higher doses for VMAT. Mean dose-volume histograms for head-CTV and brain-CTV are shown in Figure 3. VMAT plans came across with the highest V_30 Gy_ for head-CTV for all patients, except for the one with the smallest CTV volume where VMAT was very similar to the 2F—but higher than the 3F—techniques. The 3F PBT technique had the lowest head-CTV V_30 Gy_ for all patients, except the one with the largest CTV volume. Which of the 2 2F techniques that gave the lowest V_30 Gy_ differed from case to case (Figure 3 (C)).

**Table 1:** Dose to brain-CTV and head-CTV (median values with range).

	VMAT	Three beams proton: 3F (Gy RBE)	Two beams proton: 2F-L (Gy RBE)	Two beams proton: 2F-IV (Gy RBE)	Likely clinical importance
Brain-CTV, V_30 Gy_ (%)	5.1 (1.7-6.6)	3.1 (1.3-4.2)	3.3 (1.4-4.2)	3.4 (1.4-4.8)	Low
Head-CTV, V_30 Gy_ (%)	3.5 (1.0-5.0)	2.1 (0.8-3.0)	2.2 (0.8-3.0)	2.3 (1.0-3.8)	Low
Brain-CTV, D_mean_	4.8 (3.2-7.1)	3.9 (2.0-4.8)	3.1 (1.7-4.1)	4.1 (2.0-5.7)	Low
Head-CTV, D_mean_	4.7 (2.2-5.8)	2.5 (1.3-3.5)	2.3 (1.2-3.1)	2.6 (1.4-4.1)	Moderate
Integral dose to brain-CTV, Gy × cm^3^	7013 (3400-9559)	5263 (2722-7341)	4428 (2476-5987)	5476 (2882-8096)	Low
Integral dose to head-CTV, Gy × cm^3^	17 813 (7040-22 118)	9526 (4009-13 394)	8979 (3874-12 267)	10 195 (4608-14 886)	Moderate
Integral dose to brain-CTV, reduction in % compared with photon	NA	26.1% (−2.5 to 51.4)	39.4% (20.9-59.8)	21.7% (−18.1 to 44.8)	Low
Integral dose to head-CTV, reduction in % compared with photon	NA	43.4 (34.4-57.6)	45.9 (41.1-60.0)	38.5 (27.9-54.3)	Moderate

**Abbreviations:** 2F, 2-field proton technique; 3F, 3-field proton technique; cc: cubic centimeters; CTV, clinical target volume; Gy, Gray; NA, not applicable; RBE, relative biological effectiveness; V_30 Gy_, volume receiving 30 Gy; VMAT, volumetric modulated arc therapy.

For head-CTV mean dose, a reduction of >20% was seen for all 15 patients with all PBT techniques compared to the VMAT plan (Figure 2A and Supplementary Table 3). A reduction of >20% for head-CTV V_30 Gy_ was seen for 13 patients with the 3F technique and 11 and 12 patients with the 2F techniques compared to the VMAT plans (Figure 2C and Supplementary Table 3). For brain-CTV, mean dose with the 2F-L technique showed a reduction of >20% (range 21.0%-59.7%) for all 15 patients, compared to VMAT (Figure 2B and Supplementary Table 3). For the 3F technique, >20% dose reduction was seen in 10 of 15 patients, whereas for the 2F-IV technique, it was seen in 8 patients. For V30Gy (RBE) for the brain-CTV volume, the 3F technique gave a dose reduction of >20% for 14 of 15 patients, 2F-L for 11 patients, and 2F-IV for 10 patients (Figure 2D and Supplementary Table 3).

Volumes receiving 50%, 25%, and 10% of the prescribed dose, that is, V_50%_, V_25%_, and V_10%_, respectively, were lower for all 3 PBT techniques compared to VMAT plans, whereas the volume receiving 95% of the prescribed dose (V_95%_) was not (Supplementary Figure 1). When comparing PBT techniques, the 2F-IV technique gave the highest V_25%_ for all but 1 patient, and 3F the highest V_10%_ for all patients. The integral dose for brain-CTV had a median reduction of 29.1% comparing the 3 proton techniques to the VMAT plans, with a range from −18.1% (proton plans highest) to 59.8% (VMAT plan highest). For head-CTV, the corresponding range was 27.9% to 60.0%, with a median reduction of 42.6% compared to VMAT (Table 1 and Figure 2).

**Figure 4:**
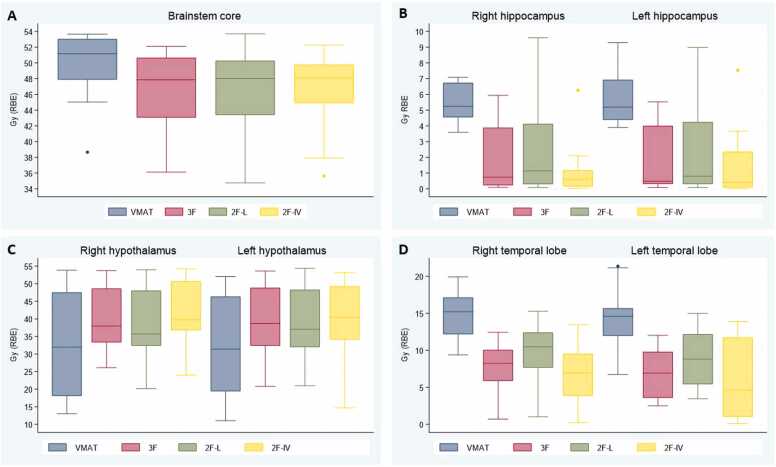
Box plots describing doses to organs at risk for brainstem core (A), hippocampi (B), hypothalami (C), and temporal lobes (D) for photon, 3-field (3 F) proton, and 2 2-field (2F-L and 2F-IV) proton techniques, respectively. Hippocampal dose to 40% of the organ volume is given, and for brainstem dose to D_0.03cm^3^_ (dose to 0.03 cm^3^). For all other organs, mean doses are shown. Horizontal lines of the boxes represent the median and the first and third quartiles. Whiskers show the smallest and largest values within 1.5 times the interquartile range, whereas data points outside the whiskers are displayed as individual dots. 2F-IV: 2-field proton technique with 2 lateral fields; 2F-L: 2-field proton technique with 2 lateral fields; 3 F: 3-field proton technique; Gy: Gray; RBE: Relative Biological Effectiveness; VMAT: Volumetric Modulated Arc Therapy.

Doses to the hippocampi were lowest using the PBT 2F-IV technique, with a median D_40%_ to the right and left hippocampus of 0.6 and 0.4 Gy (RBE), respectively. Corresponding values for the 3F technique were 0.7 to the right and 0.5 Gy (RBE) to the left hippocampus, compared to 1.1 and 0.8 Gy (RBE) for the 2F-L technique and 5.2 Gy to both hippocampi for photons. These differences were significant for both hippocampi in pair-wise comparisons between the VMAT and the 3 PBT plans and also when comparing the 2F-L with the 2F-IV technique (Supplementary Table 4/Figure 4).

For brainstem core, median D_0.03cm^3^_ was significantly higher for photon (51.2 Gy) compared to PBT plans (47.9 Gy (RBE) for 3F, 48.0 Gy (RBE) for 2F-L, and 48.1 Gy (RBE) for 2F-IV, respectively) (Figure 4 (A)). Only minor differences in median D_0.03cm^3^_ to the chiasm and optic nerves were found (Supplementary Figure 2 and Supplementary Table 2). No significant differences in D_0.03cm^3^_ were detected for the temporal lobes when comparing the 4 techniques. However, the mean dose to the temporal lobes came across as significantly different for all pair-wise comparisons between techniques, except when comparing 3F with 2F-IV bilaterally (Figure 4 (D) and Supplementary Table 4). Doses to the SC came across as very similar for the 4 treatment techniques, with median D_mean_ ranging between 52.8 and 52.9 Gy (RBE), and median D_0.03cm^3^_ 55.0 and 56.1 (Supplementary Table 2).

When comparing doses to other OARs, such as lenses, lacrimal glands, retinae, cornea, and cochlea, pair-wise comparisons between VMAT and the 3 PBT techniques showed highly significant dose reductions for all organs. For some of these OARs, there were also significant differences among the 3 PBT techniques (Supplementary Figure 2/Supplementary Table 4).

The Radiotherapy Treatment plannING study Guidelines score of this study is 100%; see Supplementary material.

## Discussion

We have shown that VMAT and all PBT techniques yielded excellent target volume coverage and acceptable doses to OARs for all 60 PitNET treatment plans. Most proton plans showed a significant dose reduction to OARs compared to corresponding VMAT plans. Hence, in that regard, protons would be preferable for most, if not all, included PitNET patients. The one exception seems to be small tumors, which are anyhow most often treated with stereotactic radiosurgery and not fractionated radiation therapy. As one would expect, OARs located near the target volume profited less from PBT techniques than more distant OARs. We speculate that the demonstrated dose reduction using PBT compared to VMAT might be clinically relevant, especially for the hippocampi and integral dose to head and brain, whereas clinical relevance for other OARs is uncertain. Our most important finding is that the optimal radiation dose distribution in patients with PitNETs is not a question of mere proton or photon therapy: optimizing proton treatment plans for the individual patient seems to be equally and even more important.

For most of our 15 patients, we demonstrated a relative dose reduction of >20% for mean dose and V30Gy to brain-CTV and head-CTV when comparing PBT techniques to VMAT. The median absolute dose reduction for mean dose to brain-CTV ranged from 0.7 to 1.7 Gy (RBE), which is low and likely of little clinical relevance. Interestingly, a dose reduction >20% was more often fulfilled for head-CTV than brain-CTV. For head CTV, the absolute dose sparing was higher, although not more than 2.1 to 2.4 Gy (RBE). Nonetheless, for PitNET patients, we argue that head-CTV is a more meaningful parameter than the more established brain-CTV, as much of the area receiving dose is located outside the brain. Secondary neoplasms may occur even in areas that have received low doses,^21^, ^23^ some argue as low as 1 to 2 Gy,^22^ often with a long latency, arguing for increased clinical importance of the dose sparing to healthy tissue demonstrated in this study. We demonstrated that the integral dose to brain-CTV and head-CTV using VMAT was higher for almost all 15 patients, whereas PBT employing 2 lateral fields was lowest for all patients. The 2F-L plans had smaller volumes receiving 10% isodose compared to all other techniques, whereas 3F plans had lower volumes for 25% isodose for all 15 patients and for most patients looking at the volume receiving 50% isodose. We suggest that an evaluation of integral dose should be part of the evaluation in cases of comparative planning, although we acknowledge that the clinical benefit remains unknown. Interestingly, our findings also suggest that the larger the target volume is, the more healthy tissue will be spared with PBT compared to VMAT.

Median D_40%_ to both hippocampi was significantly spared; 5.2 Gy using VMAT compared to 0.4 to 1.1 Gy (RBE) with the 3 PBT techniques. We speculate that this difference, although all 60 plans fulfilled dose constraints to the hippocampi as suggested by Gondi et al,^42^, ^45^ probably is clinically significant by reducing the risk of long-term cognitive impairment. PBT, especially using the 2F-IV technique, allows sparing of the dominant hippocampus, which should probably be strived for more in radiation therapy in general. Several ongoing trials evaluate neurocognitive impairment following radiation therapy, including proton therapy, and these will hopefully contribute valuable insight on the clinical importance of dose sparing of the hippocampi (NCT06036706, NCT05727605, NCT05895344, NCT03180502, NCT05190172). Based on the findings by Bachtiary et al,^46^ where no safe dose to the cochleae was found, the dose reduction to the cochleae using PBT techniques in our study was evaluated as clinically moderate. The sparing is probably especially important for patients with a long life expectancy, such as patients with PitNETs.^36^, ^47^

Temporal lobe doses were also lower with PBT compared to VMAT, corresponding to findings by others.^29^, ^48^ However, Goddard et al^29^ discussed that clinically significant dose-sparing was achievable using advanced photon planning. Furthermore, all treatment techniques fulfilled dose constraints for cochleae, lenses, retinae, corneae, and glandulae lacrimales. Although the 3 PBT techniques gave a substantial dose reduction for all these OARs compared to VMAT, the clinical relevance is most likely low (Supplementary Table 2). Dose constraints to CW and large intracerebral vessels are not firmly established; however, high doses are linked to increased risk for cerebrovascular incidents.^19^, ^20^, ^24^, ^41^ We evaluated doses to the SC as a substitute for doses to CW and large vessels.^19^, ^41^ Only minor differences between PBT techniques and VMAT, unlikely to be clinically relevant, were found. The dose differences between the 4 plans were also small for critical OARs such as the chiasm, brainstem, and optic nerves.

Although VMAT led to higher doses in most OARs, it came out superior for D_mean_ to the hypothalamus compared to PBT. Such mixed results are often seen in dosimetric studies.^39^ Mixed results were also seen when comparing different PBT techniques, for example, the 2F-IV technique generally spared contralateral OARs better compared to the other PBT techniques, at the expense of higher doses to ipsilateral OARs. Furthermore, we acknowledge that RBE and linear energy transfer (LET) uncertainties are important,^49^, ^50^, ^51^ and LET was assessed qualitatively choosing field angles considering the target volume and OAR localization. Although RBE for protons is set at 1.1, the true value is more likely variable depending on multiple factors. For PitNETs, critical structures such as the anterior optic pathways are in close proximity to the target volume, and a variable RBE leading to higher LET may bring about a higher risk of toxicity and should be taken into account in dose planning.^49^, ^50^, ^51^

The availability of PBT and whether it is reimbursed or not will inevitably affect patient selection for PBT in different countries.^52^, ^53^ The choice of selection criteria is still a matter of debate, and the clinical cost-benefit aspect of the herein demonstrated dose reduction to OARs is unclear. The as low as reasonably achievable principle often advocated to spare patients from possible toxicity,^42^ makes PBT theoretically preferable for most patients in this study. However, further studies with long-term follow-up of patients are needed to investigate tumor control, unwanted late effects, and cost issues. Whether protons yield other side effects than photons, for example, a higher frequency of symptomatic radiation-induced contrast enhancement, should also be explored.^54^, ^55^

With this study's high test number and small patient numbers, all *P*-values must be considered exploratory. Also, 4 different radiation therapy planners made the 60 treatment plans. These planners will inevitably prioritize slightly differently after fulfilling dose constraints, and this might be viewed as a weakness; however, it reflects real-world data and is, as such, a strength.

## Conclusion

In most Pit-NET patients, doses to most OARs were significantly lower with PBT techniques than VMAT. Although conclusions cannot be drawn, we speculate that the demonstrated dose reduction to some OARs, as well as the reduced integral dose, may manifest as a clinically significant toxicity reduction. Proton therapy led to a target coverage equivalent to VMAT, and it differed in which PBT technique was the best in sparing doses to OARs. We have thereby demonstrated that radiation therapy in Pit-NET patients is not a simple question of photons or protons: individualized planning based on size and localization of the target volume is the important task, and we conclude that one size does not fit all for proton planning of patients with PitNET.

## Ethics

The study was approved by the institutional data protection office. All patients signed a written consent form prior to inclusion.

## Funding

This treatment planning study has received financial support from the South-Eastern Norway Regional Health Authority, the Norwegian Cancer Society, and the Larvik Cancer Society.

## Author Contributions

Liv Cathrine Heggebø: Conceptualization, Methodology, Formal analysis, Investigation, Writing- Original draft. Frank Leonel Bello Garrote: Methodology, Formal analysis, Investigation, Writing- Review and Editing. Lars Fredrik Fjæra: Methodology, Formal analysis, Investigation, Writing- Review and Editing. Maziar Hervani: Methodology, Investigation, Writing- Review and Editing. Christina Ramberg: Methodology, Writing- Review and Editing. Andreas Ottestad: Methodology, Investigation, Writing- Review and Editing. Hanne Blakstad: Writing- Review and Editing. Trine Martens: Methodology, Writing- Review and Editing. Corina S. Rueegg: Methodology, Investigation, Validation, Writing- Review and Editing. Magnus Gustavsson: Supervision, Writing- Review and Editing. Thomas Henry: Supervision, Writing- Review and Editing. Malin Blomstrand: Supervision, Writing- Review and Editing. Taran Paulsen Hellebust: Conceptualization, Methodology, Writing- Review and Editing. Petter Brandal: Conceptualization, Methodology, Writing- Original draft, Supervision, Funding acquisition.

## Declaration of Conflicts of Interest

The authors declare that they have no known competing financial interests or personal relationships that could have appeared to influence the work reported in this paper.

## Data availability

Data material is available at the study center upon request.

## Appendix A Supplementary material

.

Supplementary Figure 1: Graphs illustrating the size of the volume for each of the 15 patients receiving 95% (A), 50% (B), 25% (C), and 10% (D) of the 54 Gy Relative Biological Effectiveness isodose, comparing the 4 treatment techniques. 2F-IV: 2-field proton technique with 2 lateral fields; 2F-L: 2-field proton technique with 2 lateral fields; 3F: 3-field proton technique; VMAT: Volumetric Modulated Arc Therapy

.

Supplementary Figure 2: Box plots describing doses to chiasm (A), optic nerves (B), lenses (C), cornea (D), retinae (E), and lacrimal glands (F) for photon, 3-field (3F) proton, and 2 2-field (2F-L and 2F-IV) proton techniques, respectively. For lacrimal glands, the mean doses are shown. Doses to D_0.03cm__^3^_ (dose to 0.03 cm^3^) are shown for all other organs. Horizontal lines of the boxes represent the median and the first and third quartiles. Whiskers show the smallest and largest values within 1.5 times the interquartile range, whereas data points outside the whiskers are displayed as individual dots. 2F-IV: 2-field proton technique with 2 lateral fields; 2F-L: 2-field proton technique with 2 lateral fields; 3F: 3-field proton technique; Gy: Gray; RBE: Relative Biological Effectiveness; VMAT: Volumetric Modulated Arc Therapy

## Footnotes

Appendix A Supplementary data associated with this article can be found in the online version at doi:10.1016/j.ijpt.2025.101194.
