# Supplementary material for: Protons or Photons in Pituitary Neuroendocrine Tumors—That Is Not the Question
Source: Int J Part Ther. 2025 Jun 18;17:101194. doi: 10.1016/j.ijpt.2025.101194 (PMC12268004; doi:10.1016/j.ijpt.2025.101194)
Supplement: Supplementary file 2 — Supplementary material [file mmc2.docx]

Supplementary Table 1: Patient characteristics

| **Patient** | **Gender** | **Adenoma subtype** | **Tumor size at time of radiotherapy (in millimeters)** |
| --- | --- | --- | --- |
| 1 | Female | Prolactinoma | 20x15x19 |
| 2 | Male | Somatotroph adenoma | 13x12x14 |
| 3 | Male | Non-functioning adenoma | 21x26x16 |
| 4 | Female | Non-functioning adenoma | 22x16x21 |
| 5 | Male | Non-functioning adenoma | 33x25x19 |
| 6 | Female | Non-functioning adenoma | 26x25x29 |
| 7 | Female | Non-functioning adenoma | 24x15x26 |
| 8 | Male | Somatotroph adenoma | 11x9x8 |
| 9 | Male | Non-functioning adenoma | 39x27x25 |
| 10 | Female | Non-functioning adenoma | 29x26x17 |
| 11 | Male | Non-functioning adenoma | 18x13x16 |
| 12 | Male | Somatotroph adenoma | 12x6x11 |
| 13 | Female | Thyreotroph adenoma | 18x19x16 |
| 14 | Female | Non-functioning adenoma | 22x22x12 |
| 15 | Male | Non-functioning adenoma | 32x38x21 |
