# Supplementary material for: Protons or Photons in Pituitary Neuroendocrine Tumors—That Is Not the Question
Source: Int J Part Ther. 2025 Jun 18;17:101194. doi: 10.1016/j.ijpt.2025.101194 (PMC12268004; doi:10.1016/j.ijpt.2025.101194)
Supplement: Supplementary file 3 — Supplementary material [file mmc3.docx]

Supplementary Table 2: Median (range) doses to different volumes and organs

|  | **VMAT** | **3 beams proton:**  **3F (Gy RBE)** | **2 beams proton:**  **2F-L (Gy RBE)** | **2 beams proton:**  **2F-IV (Gy RBE)** | **Likely clinical importance** |
| --- | --- | --- | --- | --- | --- |
| **Brainstem core, D_0.03cc_** | 51.2 (38.7-53.7) | 47.9 (36.1-52.1) | 48.0 (34.6-53.7) | 48.1 (35.6-52.2) | None |
| **Chiasm, D_0.03cc_** | 53.1 (50.6-53.9) | 52.7 (48.0-53.3) | 52.8 (48.9-54.0) | 52.8 (49.8-54.0) | None |
| **Right optic nerve, D_0.03cc_** | 52.9 (30.5-53.8) | 52.6 (32.8-53.7) | 52.8 (32.5-54.0) | 52.3 (27.7-53.7) | None |
| **Left optic nerve, D_0.03cc_** | 52.8 (39.4-53.8) | 52.4 (29.8-53.7) | 52.6 (27.3-53.7) | 52.4 (28.0-53.5) | None |
| **Right lens, D_0.03cc_** | 2.9 (2.1-6.9) | 0.0 (0.0-0.0) | 0.0 (0.0-0.0) | 0.0 (0.0-0.0) | Low |
| **Left lens, D_0.03cc_** | 3.3 (2.3-6.2) | 0.0 (0.0-0.0) | 0.0 (0.0-0.0) | 0.0 (0.0-0.0) | Low |
| **Right cornea, D_0.03cc_** | 6.8 (3.6-12.6) | 0.0 (0.0-0.0) | 0.0 (0.0-0.0) | 0.0 (0.0-0.0) | Low |
| **Left cornea, D_0.03cc_** | 6.4 (3.2-9.2) | 0.0 (0.0-0.0) | 0.0 (0.0-0.0) | 0.0 (0.0-0.0) | Low |
| **Right retina, D_0.03cc_** | 12.8 (6.8-18.0) | 0.9 (0.0-3.6) | 1.0 (0.0-4.1) | 0.3 (0.0-6.1) | Low |
| **Left retina, D_0.03cc_** | 12.3 (6.3-17.8) | 0.6 (0.0-3.6) | 0.8 (0.0-4.6) | 0.3 (0.0-5.5) | Low |
| **Right lacrimal gland, D_mean_** | 8.9 (0.7-15.2) | 0.1 (0.0-0.8) | 0.1 (0.0-1.0) | 0.0 (0.0-2.4) | Low |
| **Left lacrimal gland, D_mean_** | 5.9 (2.3-13.6) | 0.1 (0.0-0.9) | 0.1 (0.0-1.9) | 0.0 (0.0-2.8) | Low |
| **Right cochlea, D_mean_** | 21.3 (6.5-31.3) | 1.6 (0.0-5.9) | 2.9 (0.0-8.3) | 0.8 (0.0-7.2) | Moderate |
| **Left cochlea, D_mean_** | 21.5 (5.4-34.6) | 1.0 (0.0-10.4) | 1.5 (0.0-16.6) | 0.7 (0.0-15.1) | Moderate |
| **Right hippocampus, D_40%_** | 5.2 (3.6-7.1) | 0.7 (0.1-5.9) | 1.1 (0.1-9.6) | 0.6 (0.1-6.3) | Moderate |
| **Left hippocampus, D_40%_** | 5.2 (3.9-9.3) | 0.5 (0.1-5.5) | 0.8 (0.1-9.0) | 0.4 (0.0-7.5) | Moderate |
| **Right hypothalamus, D_mean_** | 32.0 (13.0-53.9) | 38.0 (26.1-53.7) | 35.7 (20.1-54.0) | 39.8 (24.0-54.2) | None |
| **Left hypothalamus, D_mean_** | 31.4 (11.1-52.0) | 38.7 (20.8-53.6) | 37.0 (21.0-54.4) | 40.5 (14.7-53.1) | None |
| **Right temporal lobe, D_0.03cc_** | 54.5 (37.8-56.5) | 55.3 (23.8-56.3) | 55.1 (27.7-56.5) | 55.9 (21.5-56.4) | None |
| **Left temporal lobe, D_0.03cc_** | 54.2 (31.7-55.9) | 54.9 (21.1-56.8) | 54.8 (29.2-56.5) | 55.4 (17.3-56.3) | None |
| **Right temporal lobe, D_mean_** | 15.2 (9.4-19.9) | 8.2 (0.7-12.4) | 10.5 (1.0-15.3) | 6.9 (0.2-13.5) | Low |
| **Left temporal lobe, D_mean_** | 14.6 (6.7-21.4) | 6.9 (2.5-12.0) | 8.8 (3.5-15.0) | 4.6 (0.1-13.9) | Low |
| **Supracellar cistern, D_mean_** | 52.8 (45.0-53.8) | 52.9 (43.7-53.8) | 52.9 (43.7-53.8) | 52.8 (44.5-54.0) | None |
| **Supracellar cistern, D_0.03cc_** | 55.0 (54.4-56.0) | 56.1 (55.3-57.4) | 56.1 (55.3-57.4) | 56.0 (55.4-57.3) | None |
| **CTV D_2%_** | 55.0 (54.7-55.7) | 55.9 (55.1-56.7) | 55.8 (55.2-56.8) | 56.0 (55.2-56.5) | None |
| **CTV D_99%_** | 52.7 (52.2-53.2) | 52.1 (51.8-53.1) | 52.2 (51.8-53.0) | 52.3 (51.8-53.1) | None |
| 2F: 2-field proton technique; 3F: 3-field proton technique; cc: cubic centimeters; CTV: clinical target volume; D_0.03cc_: maximum dose as defined by NRG Oncology clinical trials –dose to 0.03 cubic centimeters; D_2%_: dose received by 2% of a given volume; D_40%_: dose received by 40% of a given volume; D_99%_: dose received by 99% of a given volume D_mean_: mean dose; D_median_: median dose; Gy: Gray; RBE: Relative Biological Effectiveness; V_30Gy_: volume receiving 30 Gy; VMAT: Volumetric Modulated Arc Therapy | | | | | |
