# Supplementary material for: Protons or Photons in Pituitary Neuroendocrine Tumors—That Is Not the Question
Source: Int J Part Ther. 2025 Jun 18;17:101194. doi: 10.1016/j.ijpt.2025.101194 (PMC12268004; doi:10.1016/j.ijpt.2025.101194)
Supplement: Supplementary file 4 — Supplementary material [file mmc4.docx]

Supplementary Table 3: Mean doses and V_30Gy_ for brain-CTV and head-CTV for all 15 VMAT plans, and absolute and relative dose reductions for the 45 proton plans compared to the VMAT plans

|  | Brain-CTV mean dose, Gy (RBE) | | | | Brain-CTV V30Gy (RBE) | | | | Head-CTV mean dose, Gy (RBE) | | | | Head-CTV V30Gy (RBE) | | | |
| --- | --- | --- | --- | --- | --- | --- | --- | --- | --- | --- | --- | --- | --- | --- | --- | --- |
|  | VMAT | 3F | 2F-L | 2F-IV | VMAT | 3F | 2F-L | 2F-IV | VMAT | 3F | 2F-L | 2F-IV | VMAT | 3F | 2F-L | 2F-IV |
| Patient 1 | 4,0 | 1,3  **(34 %)** | 1,6  **(40%)** | 1.3  **(31%)** | 2.7 | 0.9  **(32%)** | 0.7  **(26%)** | 0.7  **(26%)** | 3.7 | 1.7  **(48%)** | 1.8  **(50%)** | 1.7  **(47%)** | 2.0 | 0.6  **(27%)** | 0.4  (19%) | 0.5  **(22%)** |
| Patient 2 | 3.2 | 1.2  **(37%)** | 1.5  **(46%)** | 1.2  **(37%)** | 1,7 | 0.4  **(25%)** | 0.3  (17%) | 0.3  (19%) | 2.8 | 1.4  **(51%)** | 1.5  **(55%)** | 1.4  **(51%)** | 1.2 | 0.2  (17%) | 0.2  (10%) | 0.2  (16%) |
| Patient 3 | 7.1 | 3.1  **(44%)** | 3.8  **(54%)** | 3.0  **(42%)** | 6.3 | 2.9  **(46%)** | 2.5  **(38%)** | 2.8  **(44%)** | 5.4 | 2.9  **(54%)** | 3.1  **(57%)** | 3.0  **(54%)** | 3.5 | 1.5  **(43%)** | 1.3  **(37%)** | 1.5  **(42%)** |
| Patient 4 | 6.5 | 3.3  **(51%)** | 3.9  **(60%)** | 2.9  **(45%)** | 5.6 | 3.0  **(54%)** | 2.7  **(49%)** | 2.7  **(49%)** | 5.0 | 2.9  **(58%)** | 3.0  **(60%)** | 2.6  **(53%)** | 3.3 | 1.5  **(47%)** | 1.4  **(42%)** | 1.3  **(39%)** |
| Patient 5 | 6.0 | 2.4  **(40%)** | 2.9  **(48%)** | 2.0  **(34%)** | 6.6 | 3.3  **(50%)** | 2.8  **(42%)** | 2.2  **(48%)** | 4.4 | 2.2  **(49%)** | 2.3  **(53%)** | 2.0  **(46%)** | 4.3 | 2.4  **(55%)** | 2.1  **(50%)** | 2.3  **(54%)** |
| Patient 6 | 5.0 | 0.2  (3%) | 1.2  **(24%)** | -0.7  (-15%) | 4.5 | 0.6  (15%) | 0.7  (16%) | -0.3  (-7%) | 5.8 | 2.3  **(39%)** | 2.7  **(46%)** | 1.7  **(30%)** | 4.3 | 1.3  **(29%)** | 1.3  **(30%)** | 0.5  (13%) |
| Patient 7 | 5.8 | 1.5  **(26%)** | 1.7  **(29%)** | 1.2  **(22%)** | 5.5 | 2.2  **(41%)** | 1.8  **(34%)** | 1.6  **(30%)** | 4.9 | 2.1  **(43%)** | 2.1  **(43%)** | 1.8  **(38%)** | 4.0 | 1.8  **(45%)** | 1.5  **(37%)** | 1.4  **(36%)** |
| Patient 8 | 3.3 | 0.6  **(20%)** | 0.7  **(23%)** | 0.4  (12%) | 1.9 | 0.4  **(22%)** | 0.4  (19%) | 0.1  (8%) | 2.2 | 0.9  **(43%)** | 1.0  **(45%)** | 0.7  **(35%)** | 1.0 | 0.2  (18%) | 0.2  (16%) | 0  (-2%) |
| Patient 9 | 4.7 | 1.1  (17%) | 1.7  **(36%)** | 0.4  (6%) | 3.5 | 0.7  **(20%)** | 0.6  (17%) | 0.5  (15%) | 4.7 | 1.8  **(39%)** | 2.1  **(45%)** | 1.6  **(35%)** | 3.1 | 0.7  **(24%)** | 0.6  **(20%)** | 0.6  **(20%)** |
| Patient 10 | 4.2 | -0.1  (-2%) | 0.9  **(21%)** | -0.8  (-18%) | 4.1 | 1.0  **(24%)** | 0.8  **(21%)** | 0.6  (15%) | 5.0 | 1.7  **(34%)** | 2.0  **(41%)** | 1.4  **(28%)** | 4.2 | 1.5  **(35%)** | 1.3  **(32%)** | 1.1  **(27%)** |
| Patient 11 | 4.8 | 1.8  **(37%)** | 2.2  **(46%)** | 1.7  **(35%)** | 5.2 | 2.8  **(54%)** | 2.6  **(50%)** | 2.8  **(53%)** | 3.5 | 1.7  **(47%)** | 1.8  **(51%)** | 1.6  **(47%)** | 2.9 | 1.5  **(52%)** | 1.4  **(48%)** | 1.5  **(51%)** |
| Patient 12 | 6.8 | 2.6  **(39%)** | 3.0  **(44%)** | 2.4  **(35%)** | 5.1 | 1.6  **(32%)** | 1.3  **(26%)** | 1.2  **(24%)** | 5.0 | 2.6  **(52%)** | 2.7  **(54%)** | 2.4  **(47%)** | 2.8 | 0.7  **(25%)** | 0.6  **(20%)** | 0.4  (15%) |
| Patient 13 | 4.4 | 0.3  (8%) | 1.2  **(27%)** | 0.1  (3%) | 5.2 | 1.8  **(35%)** | 1.9  **(36%)** | 1.6  **(30%)** | 4.7 | 1.7  **(35%)** | 2.0  **(42%)** | 1.6  **(35%)** | 5.0 | 1.3  **(47%)** | 1.3  **(47%)** | 1.3  **(46%)** |
| Patient 14 | 4.8 | 1.1  **(23%)** | 1.9  **(40%)** | 0.7  (14%) | 4.4 | 1.5  **(35%)** | 1.5  **(35%)** | 1.2  **(27%)** | 4.5 | 1.8  **(40%)** | 2.1  **(45%)** | 1.6  **(36%)** | 3.6 | 1.4  **(41%)** | 1.4  **(38%)** | 1.3  **(36%)** |
| Patient 15 | 5.7 | 1.1  (19%) | 2.0  **(34%)** | 0.7  (11%) | 5.6 | 1.4  **(24%)** | 1.4  **(24%)** | 1.2  **(21%)** | 5.0 | 2.0  **(39%)** | 2.2  **(44%)** | 1.9  **(39%)** | 4.0 | 1.3  **(34%)** | 1.3  **(33%)** | 1.3  **(34%)** |
| 2F-IV: 2-field proton technique with two lateral fields; 2F-L: 2-field proton technique with two lateral fields; 3F: 3-field proton technique; CTV: clinical target volume; Gy: Gray; RBE: Relative Biological Effectiveness; VMAT: Volumetric Modulated Arc Therapy. For the three proton beam techniques the relative dose reduction compared to the photon plans are calculated and shown in parenthesis. Proton plans with a greater dose reduction than 20% compared to VMAT plans are highlighted in bold. | | | | | | | | | | | | | | | | |
