# Supplementary material for: Protons or Photons in Pituitary Neuroendocrine Tumors—That Is Not the Question
Source: Int J Part Ther. 2025 Jun 18;17:101194. doi: 10.1016/j.ijpt.2025.101194 (PMC12268004; doi:10.1016/j.ijpt.2025.101194)
Supplement: Supplementary file 5 — Supplementary material [file mmc5.docx]

Supplementary Table 4: Pair-wise statistical comparisons between the different radiotherapeutic techniques for target volumes and organs at risk

|  | **VMAT vs. 3F** | **VMAT vs. 2F-L** | **VMAT vs. 2F-IV** | | **3F vs. 2F-L** | **3F vs. 2F-IV** | **2F-L vs. 2F-IV** |
| --- | --- | --- | --- | --- | --- | --- | --- |
| Brainstem core, D_0.03cc_ | **0.001** | **0.003** | **<0.001** | 0.555 | | 0.482 | 0.359 |
| Chiasm, D_0.03cc_ | **0.003** | 0.132 | 0.032 | 0.009 | | 0.213 | 0.451 |
| Right optic nerve, D_0.03cc_ | 0.252 | 0.336 | **0.005** | 0.905 | | 0.223 | 0.194 |
| Left optic nerve, D_0.03cc_ | 0.134 | 0.404 | 0.174 | 0.268 | | 0.665 | 0.155 |
| Right lens, D_0.03cc_ | **<0.001** | **<0.001** | **<0.001** | 0.591 | | 0.022 | 0.032 |
| Left lens, D_0.03cc_ | **<0.001** | **<0.001** | **<0.001** | 0.112 | | 0.104 | 0.026 |
| Right cornea, D_0.03cc_ | **<0.001** | **<0.001** | **<0.001** | 0.112 | | 0.104 | 0.026 |
| Left cornea, D_0.03cc_ | **<0.001** | **<0.001** | **<0.001** | 0.045 | | 0.022 | **0.008** |
| Right retina, D_0.03cc_ | **<0.001** | **<0.001** | **<0.001** | 0.230 | | 0.843 | 0.748 |
| Left retina, D_0.03cc_ | **<0.001** | **<0.001** | **<0.001** | 0.474 | | 0.855 | 0.545 |
| Right lacrimal gland, D_mean_ | **<0.001** | **<0.001** | **<0.001** | 0.223 | | 0.762 | 0.856 |
| Left lacrimal gland, D_mean_ | **<0.001** | **<0.001** | **<0.001** | 0.484 | | 0.563 | 0.686 |
| Right cochlea, D_mean_ | **<0.001** | **<0.001** | **<0.001** | 0.009 | | 0.339 | 0.037 |
| Left cochlea, D_mean_ | **<0.001** | **<0.001** | **<0.001** | 0.108 | | 0.777 | 0.734 |
| Right hippocampus, D_40%_ | **<0.001** | **<0.001** | **<0.001** | 0.060 | | 0.090 | **0.007** |
| Left hippocampus, D_40%_ | **<0.001** | **<0.001** | **<0.001** | 0.060 | | 0.090 | **0.007** |
| Right hypothalamus, D_mean_ | **0.003** | 0.013 | **0.005** | 0.030 | | 0.175 | 0.044 |
| Left hypothalamus, D_mean_ | **0.005** | 0.015 | 0.016 | 0.207 | | 0.575 | 0.284 |
| Right temporal lobe, D_0.03cc_ | 0.890 | 0.905 | 0.973 | 0.869 | | 0.684 | 0.917 |
| Left temporal lobe, D_0.03cc_ | 0.361 | 0.104 | 0.471 | 0.059 | | 0.972 | 0.133 |
| Right temporal lobe, D_mean_ | **<0.001** | **<0.001** | **<0.001** | **<0.001** | | 0.230 | **0.002** |
| Left temporal lobe, D_mean_ | **<0.001** | **<0.001** | **<0.001** | **<0.001** | | 0.238 | **<0.001** |
| Brain-CTV, V_30Gy_  (in percent) | **<0.001** | **<0.001** | **<0.001** | **0.003** | | **<0.001** | 0.220 |
| Head-CTV, V_30Gy_ (in percent) | **<0.001** | **<0.001** | **<0.001** | **0.001** | | **0.003** | 0.277 |
| Brain-CTV, D_mean_ | **<0.001** | **<0.001** | **0.003** | **<0.001** | | **<0.001** | **<0.001** |
| Head-CTV, D_mean_ | **<0.001** | **<0.001** | **<0.001** | **<0.001** | | **0.002** | **<0.001** |
| CTV D_2%_ | **<0.001** | **0.001** | **<0.001** | 0.977 | | 0.240 | 0.384 |
| CTV D_99%_ | **<0.001** | **<0.001** | **<0.001** | 0.760 | | 0.809 | 0.968 |
| 2F: 2-field proton technique; 3F: 3-field proton technique; CTV: clinical target volume; D_0.03cc_: maximum dose as defined by NRG Oncology clinical trials –dose to 0.03 cubic centimeters; D_2%_: dose received by 2% of a given volume; D_40%_: dose received by 40% of a given volume; D_99%_: dose received by 99% of a given volume D_mean_: mean dose; D_median_: median dose; Gy: Gray; RBE: Relative Biological Effectiveness; V_30Gy_: volume receiving 30 Gy; VMAT: Volumetric Modulated Arc Therapy: significant p-values in bold defined as 0.05 divided by 6 comparisons, and thereby significant below 0.008 | | | | | | | |
