# Supplementary figures and images for: Protons or Photons in Pituitary Neuroendocrine Tumors—That Is Not the Question
Source: Int J Part Ther. 2025 Jun 18;17:101194. doi: 10.1016/j.ijpt.2025.101194 (PMC12268004; doi:10.1016/j.ijpt.2025.101194)

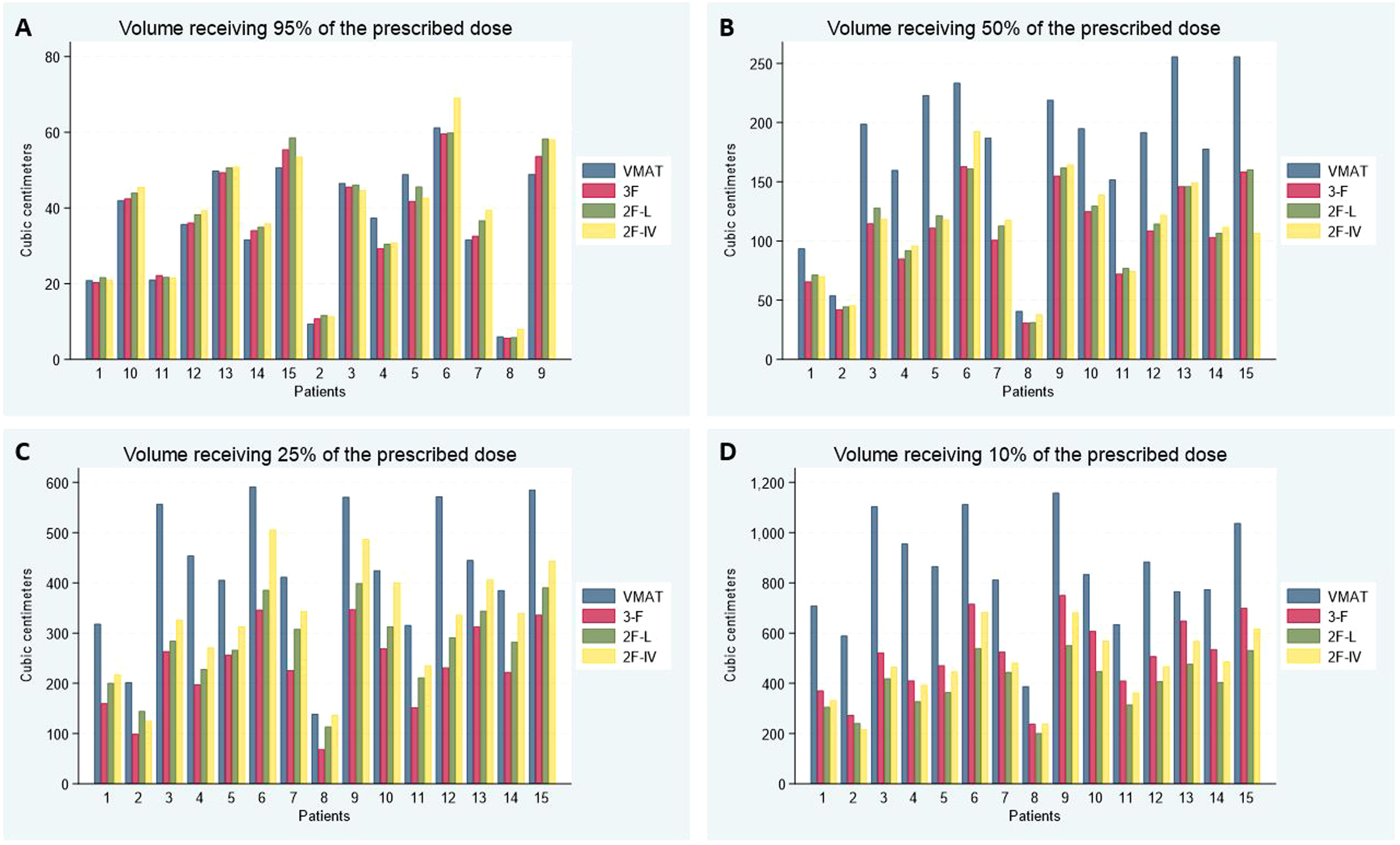

Supplement: Supplementary file 6 — Supplementary Figure 1: Graphs illustrating the size of the volume for each of the 15 patients receiving 95% (A), 50% (B), 25% (C), and 10% (D) of the 54 Gy Relative Biological Effectiveness isodose, comparing the 4 treatment techniques. 2F-IV: 2-field proton technique with 2 lateral fields; 2F-L: 2-field proton technique with 2 lateral fields; 3F: 3-field proton technique; VMAT: Volumetric Modulated Arc Therapy [file mmc6.jpg]

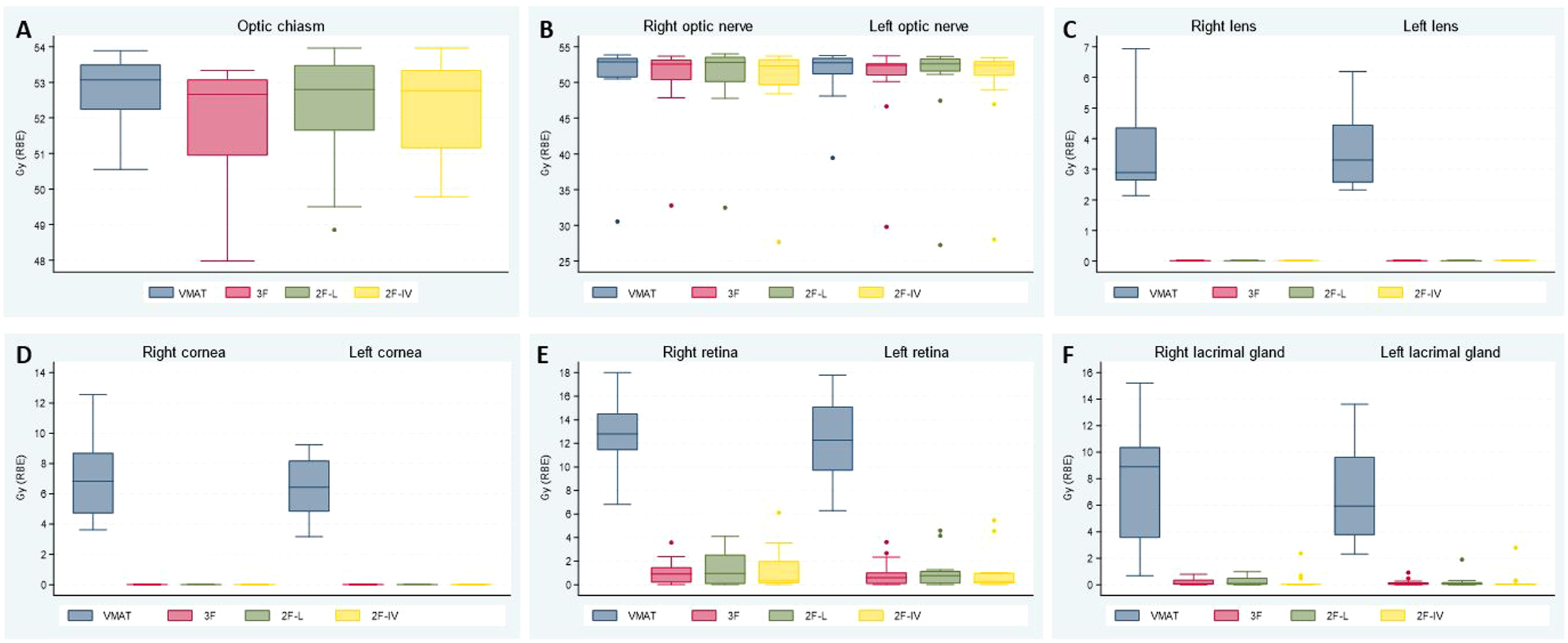

Supplement: Supplementary file 7 — Supplementary Figure 2: Box plots describing doses to chiasm (A), optic nerves (B), lenses (C), cornea (D), retinae (E), and lacrimal glands (F) for photon, 3-field (3F) proton, and 2 2-field (2F-L and 2F-IV) proton techniques, respectively. For lacrimal glands, the mean doses are shown. Doses to D0.03cm3 (dose to 0.03 cm3) are shown for all other organs. Horizontal lines of the boxes represent the median and the first and third quartiles. Whiskers show the smallest and largest values within 1.5 times the interquartile range, whereas data points outside the whiskers are displayed as individual dots. 2F-IV: 2-field proton technique with 2 lateral fields; 2F-L: 2-field proton technique with 2 lateral fields; 3F: 3-field proton technique; Gy: Gray; RBE: Relative Biological Effectiveness; VMAT: Volumetric Modulated Arc Therapy [file mmc7.jpg]
